# Chronic Powder Diet After Weaning Induces Sleep, Behavioral, Neuroanatomical, and Neurophysiological Changes in Mice

**DOI:** 10.1371/journal.pone.0143909

**Published:** 2015-12-02

**Authors:** Emiko Anegawa, Nozomu Kotorii, Yuji Ishimaru, Masashi Okuro, Noriaki Sakai, Seiji Nishino

**Affiliations:** 1 Sleep & Circadian Neurobiology Laboratory, Department of Psychiatry and Behavioral Sciences Sleep Research Center, Stanford University School of Medicine, Palo Alto, California, United States of America; 2 Department of Oral and Maxillofacial Surgery, Kurume University School of Medicine, Kurume, Fukuoka, Japan; 3 Department of Oral & Maxillofacial Surgery, Yokohama City University Graduate School of Medicine, Yokohama, Kanagawa, Japan; Osaka University, JAPAN

## Abstract

The purpose of this study is to clarify the effects of chronic powder diet feeding on sleep patterns and other physiological/anatomical changes in mice. C57BL/6 male mice were divided into two groups from weaning: a group fed with solid food (SD) and a group fed with powder food (PD), and sleep and physiological and anatomical changes were compared between the groups. PD exhibited less cranial bone structure development and a significant weight gain. Furthermore, these PD mice showed reduced number of neurogenesis in the hippocampus. Sleep analysis showed that PD induced attenuated diurnal sleep/wake rhythm, characterized by increased sleep during active period and decreased sleep during rest period. With food deprivation (FD), PD showed less enhancement of wake/locomotor activity compared to SD, indicating reduced food-seeking behavior during FD. These results suggest that powder feeding in mice results in a cluster of detrimental symptoms caused by abnormal energy metabolism and anatomical/neurological changes.

## Introduction

A series of epidemiological evidences had pointed out the importance of mastication in health and diseases. Modern diet trends allow us to change eating habits and to have soft, homogeneous, and processed diets available anytime [1, 2]. These modern foods are soft enough to be swallowed and digested quickly and thus masticatory frequencies and eating duration have decreased [2]. Although the relationship is not fully confirmed yet, there is growing awareness that the soft modern diet could lead to an increasing number of children with malocclusion and malalignment [3]. In addition to a general role in the digestive processes, mastication is likely to influence not only the development of facial structures but also systemic, mental, and physical functions of the body. A series of intervention studies in humans suggest that mastication can play an important role on energy balance through effects on appetite [4, 5], energy expenditure [5, 6] or nutrient metabolism [4, 7–9]. A recent study a has also shown interesting negative associations between body mass index (BMI) and the number of chewing cycles, as well as chewing duration before swallowing in fully dentate healthy adults [10]. In addition, several epidemiological studies in the young adults [11, 12] and the elderly [13–15] have shown a positive relationship between mastication and cognitive functions.

Human studies have implied that reduction of chewing stress by modern diet leads to a variety of unfavorable health outcomes, while animal studies have provided further insight into causal relationships and its mechanisms. Previous research using animals have verified that tooth loss or soft diets leads to changes in craniofacial morphology, increase in body weight, and impaired working and spatial memory. Many studies have shown that a soft diet caused smaller mandibular, maxillary, and cranial skeleton compared to a standard diet in rodents [16–20] and monkey [21], supporting the observations in epidemiological studies. Although soft diet-induced obesity is an inconsistent phenotype, several mechanisms related to mastication such as activation of satiety [22, 23], postprandial thermogenesis [24], and blood glucose metabolism [25], are suggested to be involved in this phenotype. It has been reported that a long-term soft diet or induced occlusal hypofunction causes poor performance on memory and learning tests [26–28]. In accordance with the behavioral tests, soft diet suppressed the neurogenesis and cell survival in the hippocampal dentate gyrus [29, 30].

Food is related to sleep/wake patterns by appetite and nutrient metabolism. Given the evidences that soft diet causes systemic changes of the body, sleep can be one of the potential concomitant factors. However, it is practically difficult to determine how modern processed foods with less hardness affect sleep-wake patterns in humans, since many internal and external factors influence sleep quality and quantity.

In the current study, we therefore evaluated chronic effects of different food consistency (solid vs. powder diet) on body weight, sleep-wake cycles, locomotor activity, core body temperature and neuroanatomical changes (i.e., neurogenesis in the hippocampus and craniofacial morphology) in mice. Sleep/wake responses to food deprivation were also evaluated and compared between the diet groups to examine if mice chronically fed with powder diet alter food-seeking behavior.

## Materials and Methods

### Animals

Twenty C57BL/6 male mice aged 25 days (immediately after weaning) were divided into two groups: solid diet group (SD, N = 10) fed a solid diet of regular rodent oval pellets (Prolab RMH 3000, PMI Nutrition International) and powder diet group (PD, N = 10) fed the same food but in powder form. All mice were maintained with solid or powder diet until they were sacrificed for immunohistochemistry. Each mouse was housed in its own individual recording cage made of polypropylene (22 cm × 16 cm × 12 cm). Animals were able to feed and move freely, and were kept under a 12:12 light–dark cycle (lights on at 07:00 h; zeitgeber time [ZT] 0), with room temperature at 23±2°C.

The study was carried out in strict accordance with the recommendations in the Guide for the Care and Use of Laboratory Animals of the National Institutes of Health. The protocol was approved by the Committee on the Ethics of Animal Experiments of the Stanford University Administrative Panel on Laboratory Animal Care. All efforts were made to minimize suffering.

### Headstage and telemetry implant surgery

At 19 weeks of age, the mice were surgically prepared for electroencephalogram (EEG) and electromyogram (EMG) recordings with a headstage attached to a cable recorder. Under 3% isoflurane anesthesia (50 mg/kg. i.p.), two of four electrodes for the EEG (stainless steel screws) were screwed into the skull 1.5 mm lateral and 1.5 mm anterior to the bregma (over the motor cortex), the other two were screwed 3 mm lateral and 1 mm anterior to the lambda (over the visual cortex), and two EMG electrodes (multistranded stainless steel wires) were inserted into the neck extensor muscle. The six leads for these electrodes were attached to one 2x3 pin header that was secured to the skull using dental acrylic.

Immediately following the electrode implantation, in order to evaluate the locomotor activity (LMA) and core body temperature (Tb), a telemetry-implanting device (G2 E-Mitter, Mini Mitter, OR) was implanted in the abdominal cavity of each mouse. After surgery, surgical wounds, animal behaviors and body weight were monitored, with a sufficient analgesic (carprofen, 3mg/kg s.c.) and antibiotic (enrofloxacin, 3mg/kg s.c.) supplied as needed.

### Data Collection in each recording session for 4 consecutive days (Baseline, Food deprivation and Recovery)

After 2 weeks of recovery period from the surgery, the mice (N = 8 for each group) were moved to specially modified Nalgene microisolator cages equipped with a low-torque slip-ring commutator (Biella Engineering, Irvine CA), and the cages were placed in a recording chamber. The next day, the headstages of the animals were connected to a slip ring commutator through a 15 to 20 cm of lightweight 6-strand shielded signal cable (NMUF6/30-4046SJ; Cooner Wire, Chatsworth, CA, USA). The commutator’s output was connected to an amplifier. The animals were allowed full freedom of movement in the recording cages.

After one week of adapting to their new environment, 24 hours of EEG, EMG, LMA and (Tb) recordings were carried out at 22 weeks of age as “Baseline”. After the Baseline, “Food Deprivation” (water available ad libitum) immediately followed for 48 hours with data collection (“Fasting Day 1" and “Fasting Day 2”), and then, the data of “Recovery” was collected for the next 24 hours. Food was removed at ZT0 on Fasting Day 1 and were re-provided at ZT0 on Recovery day. The EEG–EMG signals were acquired using Grass Instruments (West Warwick, RI) model 12 amplifiers. The EEG and EMG signals, digitally filtered (30 Hz Low Pass Filter for EEG; 10–100 Hz Band Pass Filter for EMG), were captured at 128 Hz using a sleep recording system (Vital Recorder; Kissei Comtec, Matsumoto, Japan). EEG signals collected with ipsilateral bipolar EEG electrodes placed over motor and visual cortices together with the bipolar EMG signals were used for sleep scoring.

Each mouse was housed in its own individual recording cage. Room temperature was maintained at 24 ± 1°C throughout experimentation. The cages were housed in custom-designed stainless steel cabinets with individual ventilated compartments. Food and water were available ad libitum during “Baseline” and “Recovery”. 24-hours light-dark cycle (12 hours lights on, 12 hours off) was maintained throughout the study (lights on at ZT0 at 7:00 am).

### Sleep scoring

The sleep stage of each 10-second epoch was visually scored using our standard criteria, and 50% or more of a particular state in each epoch is required to score the epoch. Briefly, wakefulness is characterized by a desynchronized, low-amplitude, mixed-frequency (>4 Hz) EEG and high EMG activity. Rhythmic theta/alpha (7–9 Hz) waves with high EMG activities may also appear. NR is characterized by a synchronized, high-amplitude, low-frequency (0.25–4 Hz) EEG and reduced EMG activity compared to wakefulness. EEG activity in REM sleep is similar to that in wakefulness with desynchronized, mixed-frequency, low-amplitude waves. EMG activity during REM sleep is reduced even further than during NR and is completely absent in many cases. Some muscle twitching may be apparent in the EMG trace during REM sleep. During REM sleep, rhythmic theta/alpha (7–9 Hz) waves with reduced EMG activity may be dominant, but not always, as this has been shown in other species [31, 32]. Sleep state changes were recorded when at least one 10-second epoch was scored as having a different sleep stage, and state episode length was defined as the length of continuous single state episode. All scoring has done by a single investigator (E. A.) blind to the animal information.

### Data analysis

#### Polygraphic data analysis

Cumulative amount of each vigilance state (wake, NR, and REM) was calculated and plotted for 24h with 1 hour interval during the light and dark periods. The mean episode duration in each vigilance state during 24h during the light period and dark period was also calculated.

The EEG power spectrum in the epoch that was scored as wake, NR and REM sleep was calculated by Fast Fourier Transform using the SleepSign analysis program. The EEG theta and delta frequency band was set at 4–9 Hz and 0.5–4.0 Hz, respectively. The EEG power (uV2) of each frequency band was averaged across 12 hr.

#### Locomotor and core temperature data analysis

Locomotor and temperature data were acquired with the telemetry receiver (Series 4000, Mini Mitter, OR) and the Vital View software (Mini Mitter, OR). Each telemetry receiver was calibrated for temperature measure before implantation. These telemetric implants were not operated with a battery, and no battery replacement or additional calibration was required during the study. Locomotor activity and temperature of each mouse were measured in each 1 hour time bin, and the mean (± SEM) values of locomotor counts (counts/ 1 hour) and temperature (°C) was plotted.

### Bromodeoxyuridine (BrdU) administration and Immunohistochemistry

Neurogenesis in the hippocampal dentate gyrus (DG) area was evaluated in the immunostained brain cross sections. At 34 weeks of age, mice in both groups received an intraperitoneal injection of BrdU, 50 mg/kg body weight (Sigma, St. Louis, MO) every 24 hours for 6 consecutive days. One day after the last injection of BrdU, the mice were deeply anesthetized (50 mg/kg pentobarbital) and perfused via the ascending aorta with heparinized 0.1M phosphate buffered saline (PBS, pH7.4) followed by 4% paraformaldehyde in PBS. Their brains were removed, post-fixed in the same fixative at 4°C overnight, and cryoprotected in 30% sucrose in PBS. The brains were then frozen, sectioned serially at 40μm in the coronal plane using a cryostat-microtome, and preserved in phosphate buffered saline (PBS) with sodium azide (Az). The specimens were then processed for BrdU immunohistochemistry with 3,3’-diaminobenzidine-tetrahydrochloride (DAB), by referencing the method described by Carroll MC [33]. Briefly, endogenous peroxidase in the specimens were blocked with 30% H_2_O_2_/ml in 0.01M PBS with 0.1% Triton X-100 (PBS-T, PH 7.4) for 20 minutes, and specimens were pretreated with 2N HCl at 37°C for 30 minutes to denature DNA. They were then placed in the blocking solution of 3% normal horse serum (NHS, Gibco®, Life Technologies, CA) in PBS-T for 1 hour (r.t) and incubated with the primary antibody, monoclonal rat anti-BrdU (0.4 g/ml; Abcam), in PBS-T with 3% NHS overnight at 4°C. After rinsed in PBS-T, those specimens were incubated with the secondary antibody, biotinylated rabbit anti-rat IgG (H+L), mouse absorbed, (1:200; Vector Laboratories, CA) in PBS-T for 2 hours (r.t), followed by use of avidin-biotin-peroxidase complex (ABC) immunoperoxidase techniques (1:100 dilution; Vectastain Elite Peroxidase System, Vector Laboratories, CA). Visualization of reaction products were accomplished by application of 0.05% DAB (Sigma-Aldrich, MO). Subsequently specimens were mounted on glass slides. Using a light microscope, the amount of stained cells, which indicate neurogenesis, in hippocampal DG in four bilateral brain cross sections (a total of four DG regions were chosen from −2.06mm to +2.54mm anteroposterior to the Bregma in each mouse) were counted blindly by one investigator (E.A.). A published mouse atlas was used to define brain segmentations [34].

### Maxillofacial skeletal morphology

**Fig 1:**
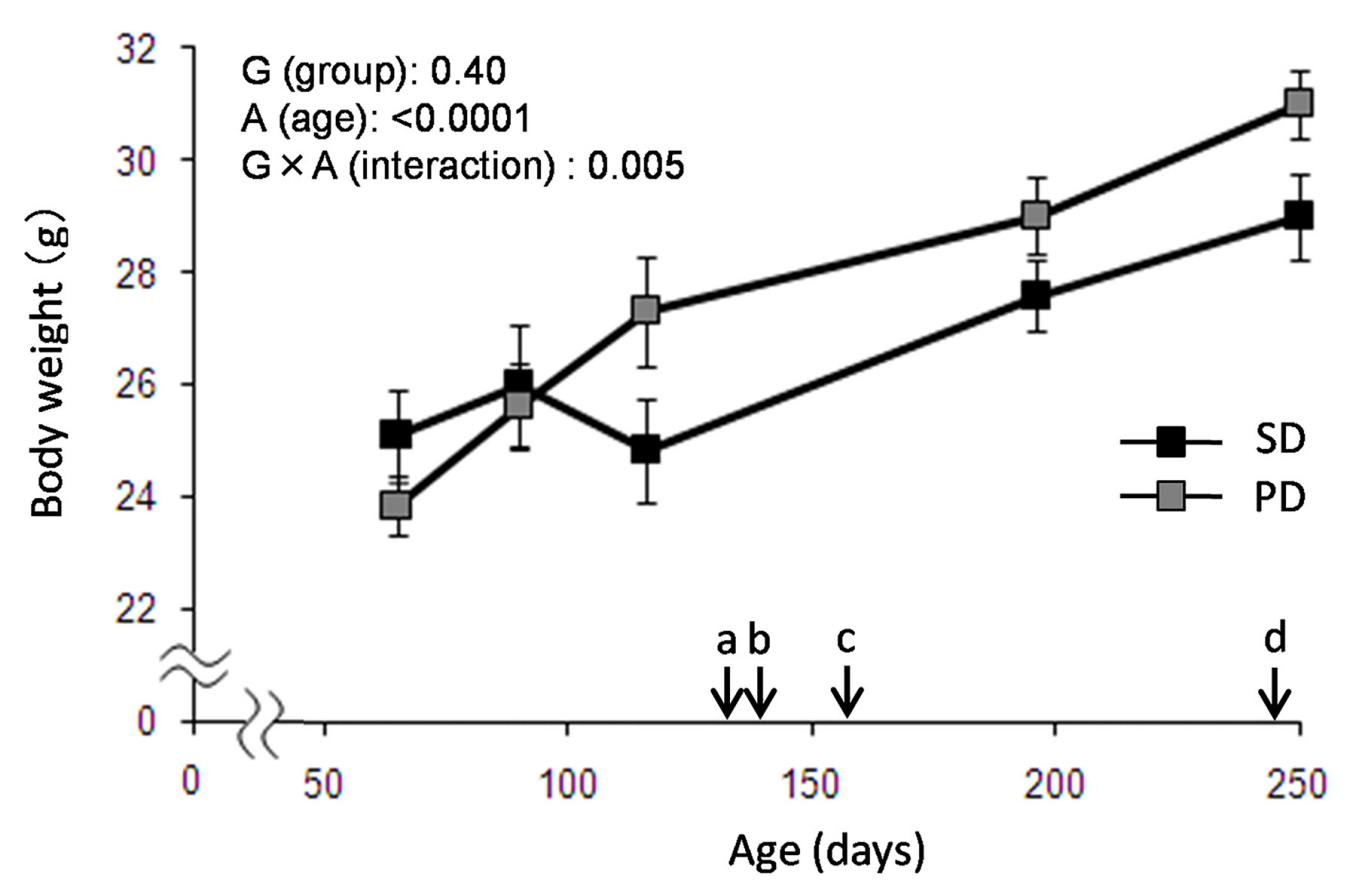
Body Weight Change in PD and SD fed mice. Body weight was measured through the experiments in both SD and PD groups (N = 8 for SD, N = 9 for PD). Each arrow indicates (a) marble burying test, (b) headstage implant for sleep recording, (c) baseline and food deprivation, and (d) BrdU injections. Data are presented as mean ± SEM.

After boiling the mouse head in hydrogen peroxide solution (H_2_O_2_), the soft tissue surrounding the skull was removed, and the form of the jaw was evaluated after drying. Measurements were performed according to a previously reported method [35]. The landmarks in craniometry were identified according to the definitions shown in S1 Fig and the following were measured: I) length of buccal alveolar, II) length of maxilla (Na-Pr), III) length of mandible (Id-Cd), IV) ramus height (Cd⊥MP), V) gonial angle (Fig 1). To measure length, a vernier caliper was used, and to measure the angle, a protractor and ruler were used. Each measurement was made three times by one investigator (E.A.) from which the average was taken. Furthermore, for the measurements of the length, height, and angle of the mandible, the average of the left and right values were used.

### Marble burying test

The marble burying test was used to evaluate the anxiety behavior in SD and PD mice (N = 10 each) at 18 week olds. The marble burying test was carried out by placing a single mouse in a polypropylene cage (22 cm × 16 cm × 12 cm) containing 12 glass marbles (1.5 cm diameter) evenly spaced on 3 cm deep sawdust. No food or water was present during the observation period. Mice have a tendency to bury objects, such as glass marbles, present in their environment. The cage was covered with a metal grid, and the number of marbles that are at least two-thirds covered was counted after 30 min. The number of marbles covered during the test has been shown to be an index of increased anxiety behavior [36, 37].

### Statistical analysis

Effects of diet on age-dependent body weight, time dependent sleep parameter changes, locomotor activity and core body temperature changes, were evaluated by repeated 2-way ANOVA (diet group, time, group x time). Effects on sleep amounts, mean episode durations, EEG frequency power and anxiety levels between diet groups were analyzed using student’s t-test. The differences between the neurogenesis of the groups in the hippocampal neurons were evaluated with the student’s t-test in each segmentation. P values less than 0.05 (two-tailed) was considered to be statistically significant.

## Results

### Morphological changes of craniofacial growth

Maxillomandibular bone growth in the PD was attenuated compared to those in the SD group, and the result is shown in Table 1. Four maxillomandibular bone structures were significantly smaller in the PD group than the SD group. Ramus height, which is proportional to mandibular bone size, showed the biggest significant difference of all measurements (t = 5.61, p<0.0001). There was no difference, in the gonial angle (t = 1.97, p = 0.069) using t-test with Bonferroni adjustment. The growth retardation of maxillomandibular structures observed in this study is consistent with the previous reports [16–21].

**Table 1:** The maxillomandibular bone size between the groups.

	Solid (N = 8)	Powder (N = 8)	*P* value*
	Mean ± SEM	Mean ± SEM	
I) length of buccal alveolar (mm)	5.94 ± 0.08	5.39 ± 0.09	0.0004
II) length of maxilla (mm)	10.42 ± 0.15	9.46 ± 0.20	0.0016
III) length of mandible (mm)	12.86 ± 0.18	11.94 ± 0.10	0.0005
IV) ramus height (mm)	6.20 ± 0.12	5.44 ± 0.05	<0.0001
V) gonial angle (°)	102.56 ± 0.71	105.38 ± 1.24	0.069

(°): degree

* Bonferroni adjustment

### Body weight changes in solid and powder diet-fed mice

**Fig 2:**
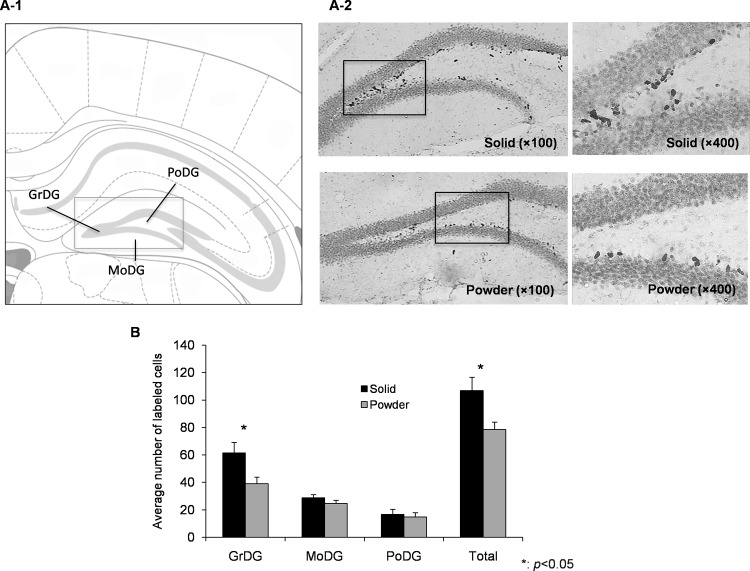
Neurogenesis in hippocampal DG. (A-1) A schematic representation in the hippocampal DG area. GrDG: granule cell layer of DG, PoDG: polymorph layer of DG, MoDG: molecular layer of the DG. (A-2) Representative photomicrographs of BrdU labeled cells in hippocampal DG at one day after the last injection of BrdU in SD (top, N = 8) and PD (bottom, N = 8) group. (B) The total number and averaged number in each area of labeled cells are presented as mean ± SEM.

Larger body weight gain was observed in PD group, compared to that in SD after 90 days old, and the weight of the PD group were about 106% of that of the SD group throughout the monitoring period (up to 250 days). Age effects (F_4,12_ = 58.00, p<0.0001) and age x diet group (F_4,12_ = 6.46, p = 0.005) effects were statistically significant with repeated two-way ANOVA (Fig 2). At 116 days, a slight and temporary weight loss unexpectedly occurred in the SD group. Although no convincing explanation for this decrease was available, food and water were provided ad libitum and no environmental change other than the regular cage change was done between 90 and 116 days.

### Neurogenesis in the hippocampal DG

Neural stem cells in the innermost layer of the hippocampal DG actively divide whereby becoming specialized as neurons. The DG is known to play a pivotal role in the formation of spatial memory through a neurotrophin-induced cellular mechanism [38]. The DG was divided into 3 layers as shown in Fig 2A: granule (GrDG), polymorph (PoDG), and molecular (MoDG). The average for each region was assessed between groups. The total number of BrdU-positive cells in the DG was 107.0 ± 9.6 in SD and 78.6 ± 5.4 in PD (t = 2.58, p = 0.022). By region, significant difference was seen in GrDG (t = 2.43, p = 0.029) but not in the PoDG (t = 0.40, p = 0.698) and MoDG (t = 1.27, p = 0.224), supporting that chronic powder feeding affects the neurogenesis in the hippocampus.

### Evaluation of the stress level in solid and powder diet-fed mice

It is known that the volume of dentate gyrus and cell proliferation are easily affected by stress [39]. To test whether different food consistency induces the stress, the marble burying test was performed. There was no significant difference in the anxiety-related behavior between the SD and PD group (t = 0.50, p = 0.62, S2 Fig). The effect of stress induced by different food consistency may be minimum on the decrease in neurogenesis in the PD group.

### Sleep/wake parameters in baseline

**Fig 3:**
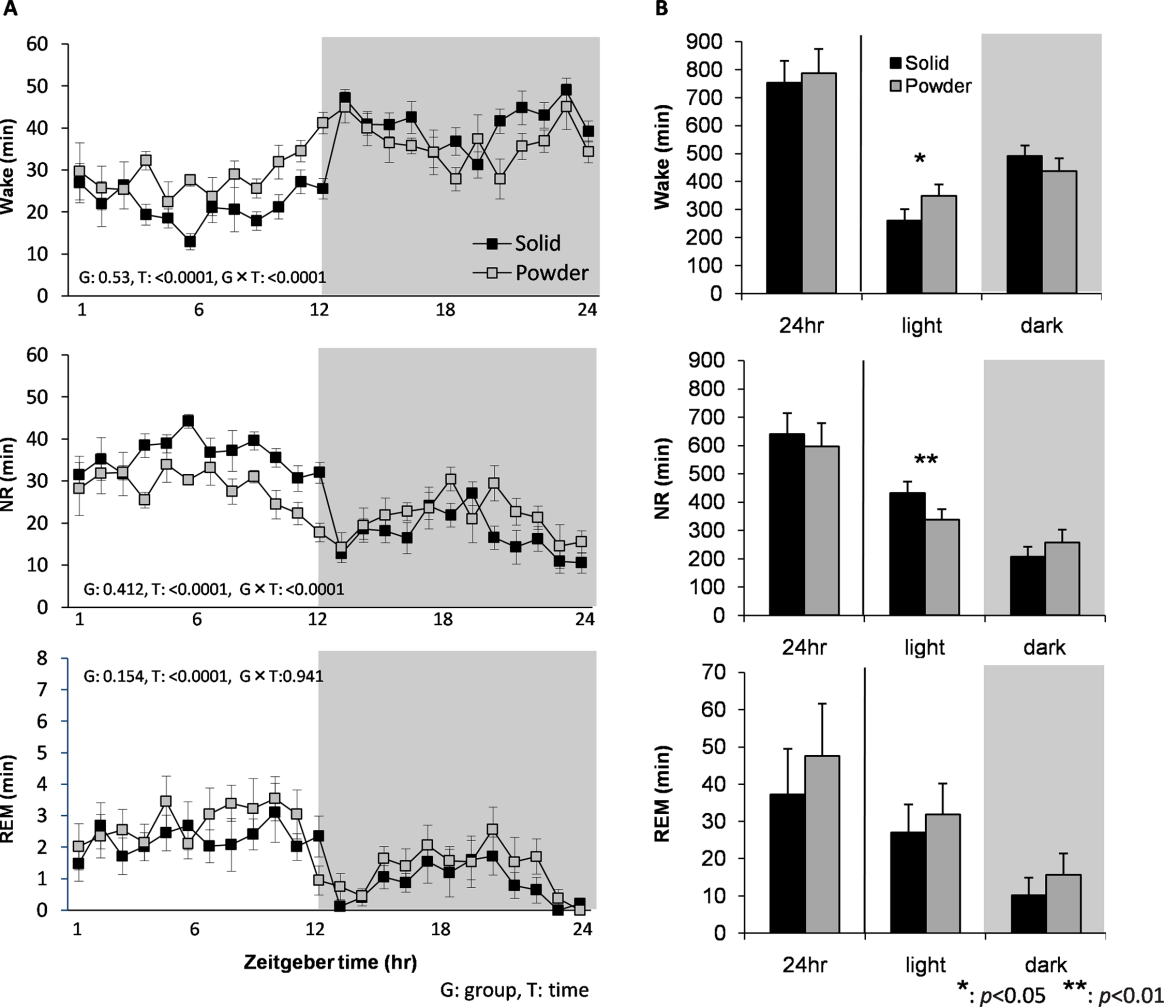
Baseline sleep/wake characterizations. (A) Time course for amount of wake (top), NR (middle), and REM sleep (bottom) over 24hr in SD and PD fed mice (N = 8 each). The gray shaded part represents the dark period. G and T are a p-value of the group and time effect, respectively. (B) Total amounts of wake, NR and REM sleep over 24hr, light, and dark periods. Data are presented as mean ± SEM.

We found significant differences in wake and NR sleep amounts between diet fed groups (F_23,299_ = 2.784, p<0.0001 and F_23,299_ = 3.007, p<0.0001, time x diet group, respectively) (Fig 3A). Wake amount of the PD in the light period was significantly larger (t = 2.782, p = 0.016), and NR amount of the light period was significantly smaller (t = 3.501, p = 0.004) than those of SD using the two-sample t-test (Fig 3B). Consequently, light/dark period ratios for wake and NR amounts were significantly reduced in PD group (t = 3.785, p = 0.003 and t = 2.198, p = 0.047, respectively), suggesting that diurnal distribution of wake and NR sleep was attenuated in PD group. In contrast, differences in amount of REM sleep between diet groups were not statistically significant (Fig 3).

**Fig 4:**
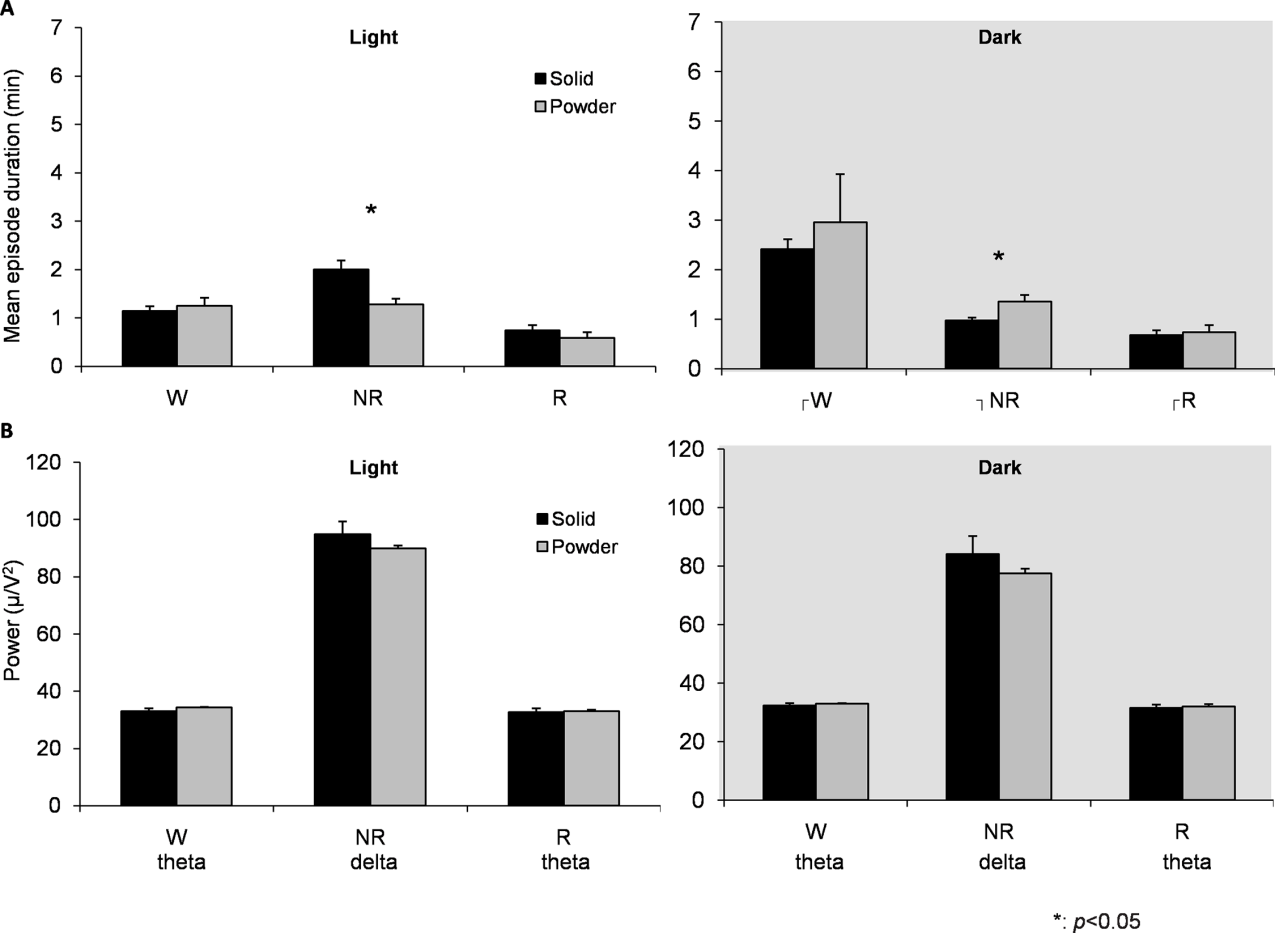
Mean episode durations and FFT power spectra at Baseline. (A) Mean episode duration of wake, NR and REM in light and dark period, (B) EEG power during wake, NR and REM. Power density in theta frequency was calculated in wake and REM sleep, while delta power density was calculated in NREM sleep. Data are presented as mean ± SEM.

The PD group had significantly shorter duration of NR episode during the light period (t = 2.918, p = 0.011) (Fig 4A), reflected in smaller NR amount during the light period. Interestingly, PD group instead had longer duration of NR episodes during the dark period (t = 2.529, p = 0.024). There were no differences in EEG power during wake, NR and REM sleep between diet groups (Fig 4B), suggesting that sleep propensity was not changed in the PD group.

### Effect of 48hr fasting on activity, temperature, and sleep at Baseline vs. Fasting Day2

**Fig 5:**
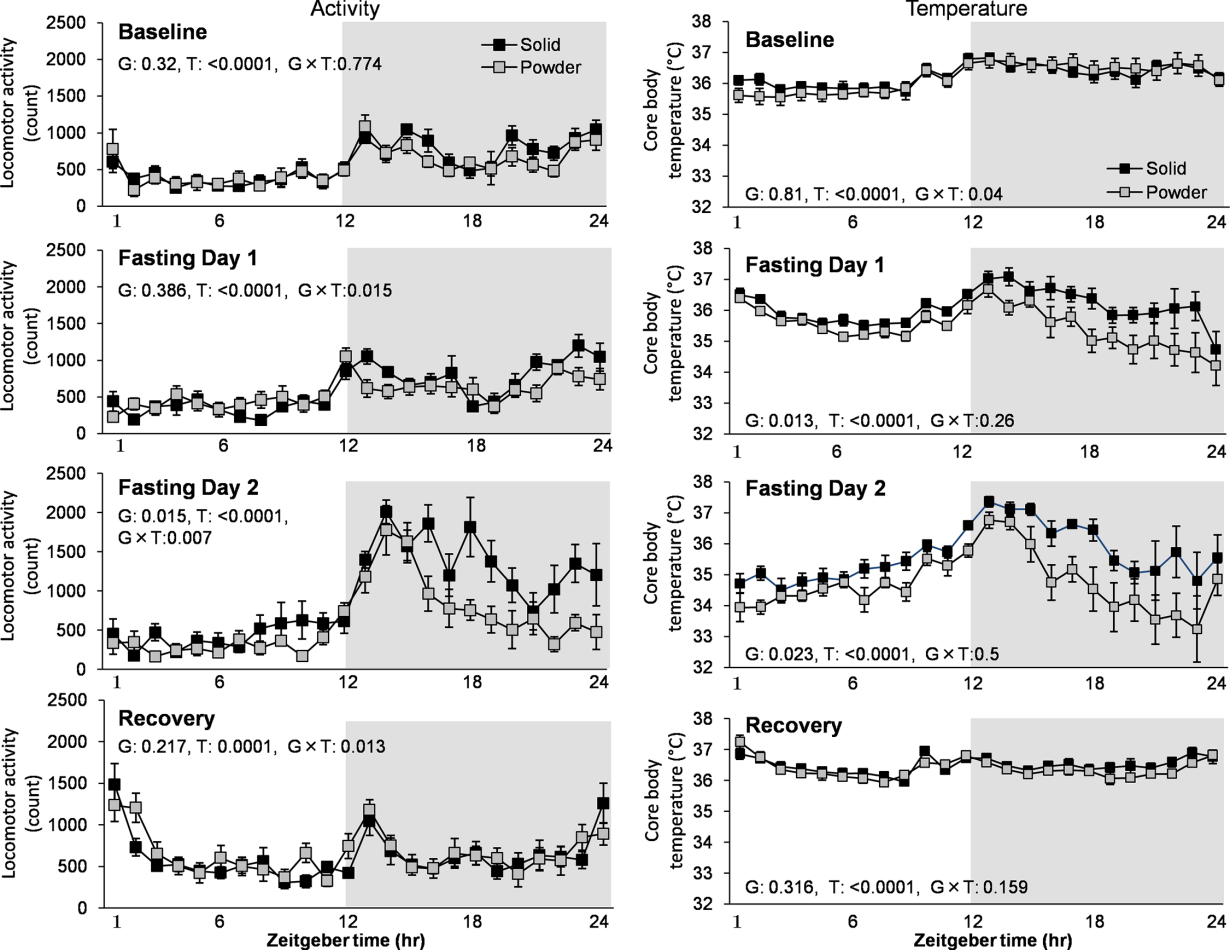
The effect of food deprivation on locomotor activity and temperature. Data collecting of locomotor activity and temperature was carried out for 4 continuous days in both groups (N = 8 each). Foods were removed at ZT0 in the Fasting Day 1 and were re-provided at ZT0 in the Recovery day. G and T are a p-values of the group and time effect, respectively. Data are presented as mean ± SEM.

Although attenuated diurnal distribution of wake and NR sleep was observed in PD group (Fig 3), there was no significant difference in LMA and temperature between groups at baseline (Fig 5). Food deprivation increased LMA during active periods associated with transient increase in Tb in the 2^nd^ day of the food deprivation in both PD and SD groups. However, the PD group exhibited less enhancement of LMA (F_1,14_ = 7.631, p = 0.015) associated with less prominent increase in body temperature (_F1,14_ = 6.533, p = 0.023) using repeated two-way ANOVA (Fig 5).

**Fig 6:**
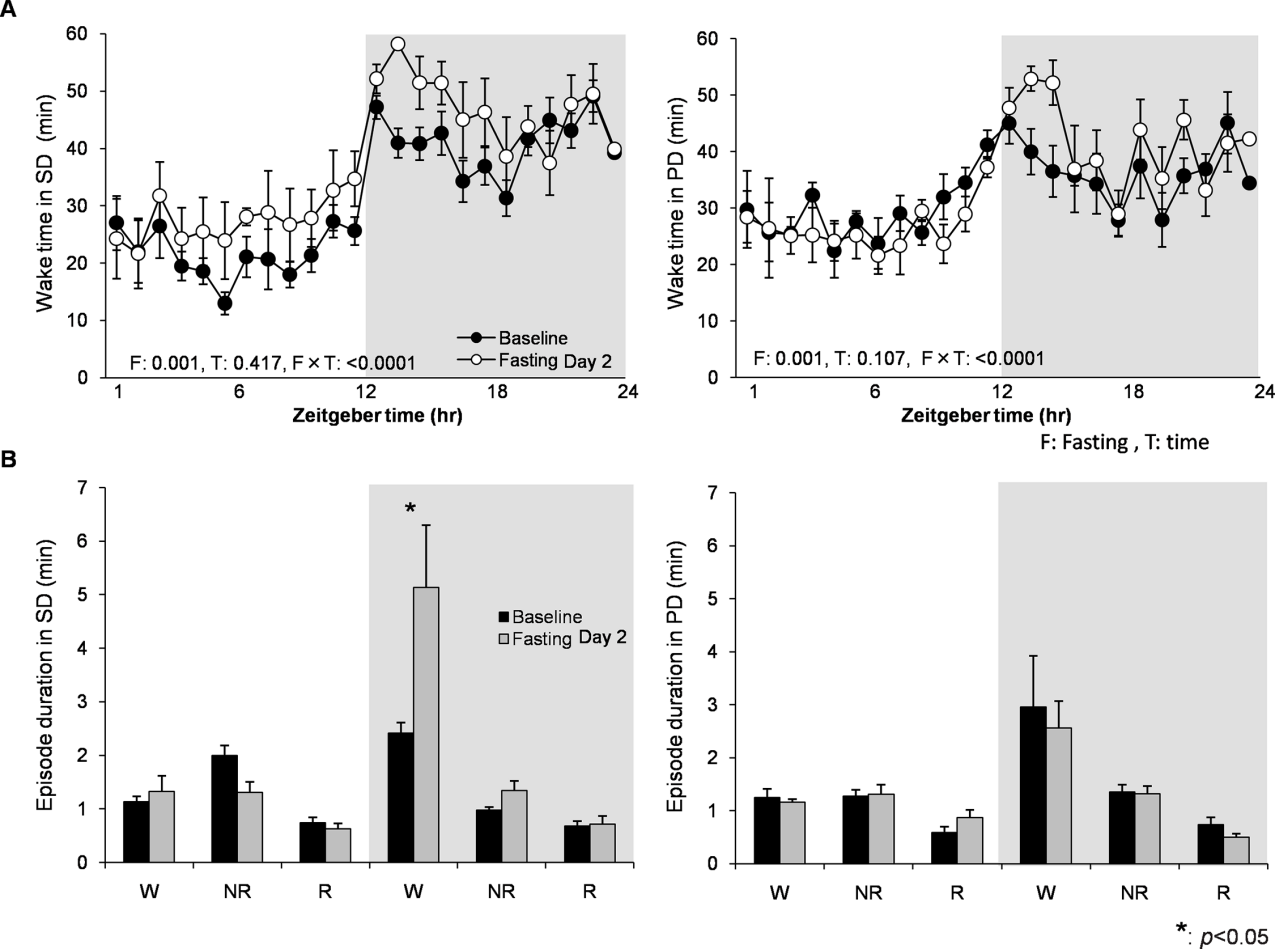
The effect of food deprivation between SD and PD diet groups at Baseline vs. Fasting Day 2. (A) Total wake amount per hour in SD (left, N = 8) and PD (right, N = 8) groups at Baseline vs. Fasting Day 2. F and T are a p-values of the fasting and time effect, respectively. (B) Mean episode duration of wake, NR, and REM at Baseline vs. Fasting Day 2. Food deprivation caused increase in wakefulness (associated with longer wake bouts) significantly in both light and dark periods in SD group, while increase in wakefulness in PD was minimum and only observed in the beginning of dark period. Data are presented as mean ± SEM.

Food deprivation significantly induced wakefulness (associated with longer wake bouts) in both light and dark periods in the 2^nd^ day in the SD group, but increase in wakefulness in the PD group was minimal and only observed in the beginning of dark period (Fig 6). This result indicates that chronic powder diet can alter food-seeking behavior.

## Discussion

In the current study, we examined the long-term effects of different food consistency (solid vs. powder diet) on sleep, behavioral, neuroanatomical and neurophysiological changes in mice from an early developmental stage.

As previously reported by several authors, we also found that powder feeding induces growth retardation of craniofacial morphology [16–21]. It is generally accepted that a decrease in mastication forces affects the structure of bone and muscles [17]. A recent study using a radiotelemetric implant showed that the duty time of the superficial masseter muscle at higher activity levels (exceeding 20% and 50% of the peak EMG) was significantly lower in the soft diet group than that in the hard diet group, indicating the decrease in muscular loading of the jaw system [19]. Enomoto et al. reported that the condylar width was significantly thinner in mice fed on soft diet for 1 week immediately following weaning and the mandibular bones was affected in terms of bone volume and length by 4 weeks [18]. These data suggest that morphological changes in craniofacial structures by powder diet can be caused by an insufficient masticatory demand during the growth period.

In the present study, the PD group showed a larger body weight than the SD group, a result consistent with some earlier reports [24, 25]. Several mechanisms related to mastication are independently or synergistically involved in soft diet-induced obesity. The soft-fed rats showed greater adiposity and attenuated postprandial thermogenesis than the hard-fed rats [24]. Hashimoto et al showed that powder diet resulted in an attenuated response to oral glucose tolerance test [25]. In addition, mastication activated the satiety center in the hypothalamus through histamine innervation [22, 23, 40]. These evidences indicate less mastication negatively affects satiety and energy metabolism. However, some studies reported no differences in body weight regardless of food consistency [16, 17, 40, 41]. This discrepancy may be explained by the differences in species, age, strains, frequencies of food refill, food consistency (hardness and water content), and duration of intervention among studies. For example, experiments with shorter interventional period, from 1 week to 4 months, result in no change in body weight [16, 17, 40, 41]. On the contrary, Wistar King A rats that fed on soft diet showed larger body weight with decreased thermogenesis after 22 week olds [24], and Wistar rats that fed on powder diet were heavier at 45 week olds or older, which are ages when abnormal glucose metabolism became prominent [25]. Interestingly, some studies reported no difference in the amount of food intake between groups at any age [24, 25, 40]. Compared to the morphological change, the body weight change likely becomes more prominent in adulthood.

Previous studies reported that mastication is important for neurogenesis and cell proliferation in the hippocampal cells [29, 30]. We also observed decreased neurogenesis in the GrDG, where neurogenesis mainly occurs, in the PD group. Onozuka et al. reported that elevated plasma corticosterone levels in the molarless condition suppressed cell proliferation of granule cells in the hippocampus [42]. In the present study, there was no difference in stress levels evaluated by the marble burying test between groups at 18 week olds. However, the involvement of chronic stress by the powder diet in the hippocampal cell loss cannot be fully excluded because only one behavioral test was applied once at the middle of the experimental period, and cognitive deficits were preferentially observed in the aged animals with molar loss [42–47]. Some studies using removal of the molar teeth or soft diet suggest the roles of acethylcholine and dopamine on synaptic density in the hippocampus [26, 27, 48]. Further studies are needed to clarify the relationship between masticatory function and the hippocampus.

With detailed sleep measures, we found that powder feeding induced attenuated diurnal sleep/wake rhythm, characterized by more sleep during active period and less sleep during the rest period. This abnormality in diurnal rhythm was caused by the change in length of sleep bouts without changing sleep pressure, indicating a less consolidated sleep in the rest period and a more consolidated sleep in the active period. It is reported that obesity is associated with increased sleep in humans [49, 50] and high-fat-fed mice [51, 52]. However, obese mice showed increased NR sleep in both light and dark, indicating increased sleep pressure and difficulties in maintaining wakefulness during the active period. Thus, the attenuated diurnal sleep/wake rhythm can not be simply explained by obesity. Considering evidences that different food consistency or disturbed masticatory functions alters the release of wake-related neurotransmitters such as acetylcholine, dopamine, histamine, and opioid, an altered sensitivity or its neurotransmission may be involved in the attenuated diurnal rhythm in the PD group [23, 26, 27, 40, 48, 53]. Timing and intensity of feeding behavior may be also involved in the attenuated rhythm. Although previous studies showed no differences in the total amount of food intake for 24 hr between different food consistencies [24, 25, 40], the PD group may intake food at different timing and intensity, leading to the different sleep/wake patterns.

It is well known that food deprivation increases wakefulness and locomotor activity for food seeking. Food deprivation influences not only amount of nutrients such as glucose, amino acids, and fatty acids but also appetite-regulating hormones such as ghrelin (stimulating appetite) and leptin (suppressing appetite) in opposite directions. It is reported that orexin/hypocretin neurons play major roles on the regulation of sleep-wake and energy homeostasis [54], and were excited by ghrelin [55] and amino acids [56] while they were suppressed by leptin and glucose [55]. In orexin neuron-ablated mice, enhancement of locomotor activity and consolidated wakefulness driven by food deprivation were absent [55]. Thus, chronic powder diet may have adverse effects on orexin neurons in order to regulate energy balance and sleep-wake patterns.

Our group recently reported that mast cell deficient (*W/W* ^*v*^) mice also showed attenuated food-seeking behavior during food deprivation [57]. Although mast cells contain numerous mediators [58, 59], we believe that histamine is another important neurotransmitter involved in this phenotype since abnormalities in hitstaminergic neurotransmission was evident in these mice [60, 61]. Orexin/histamine interactions for the control of vigilance are also well established [62, 63].

Stimulation from mastication is transferred through primary sensory afferents of the trigeminal nerve innervated by hypothalamic histaminergic neurons [22, 23, 40] and a soft diet causes a reduction in central histaminergic neurotransmissions [64]. Histamine may therefore also be involved in sleep and behavioral phenotype seen in PD mice, and further studies are required to verify this mechanism.

It is therefore important to further investigate energy metabolism and neuronal networks related to behavioral and neurological changes caused by mastication.

## Supporting Information

S1 Fig Definition of the landmarks and measurements of distances and angle. Nasion (Na): a point on the nasofrontal suture, Prosthion (Pr): the most inferior and anterior point on the alveolar process of the premaxilla, Condylion (Cd): most superodorsal point of the condylar head, Infradentable (Id): highest and most prominent point on the lower alveolar arch. Gnathion (Gn): lowest point on the mandiblar arch, Mandibular Plane (MP): Line tangent to the lower border of mandible through the Gn. Measurement items: I) length of buccal alveolar (between the maxillary second molar), II) length of maxilla (Na-Pr), III) length of mandible (Id-Cd), IV) ramus height (length of the vertical line to the MP from the highest point of condyle), V) gonial angle (the angle between MP and the line tangent to the most posterior border of mandibule through the Cd).
(TIF)

S2 Fig Anxiety-like response in PD and SD fed mice. The marble burying test was performed before the EEG/EMG surgery at 18 weeks-old (N = 10 for each group). Data are presented as mean ± SEM.
(TIF)
