# Supplementary figures and images for: Chronic Powder Diet After Weaning Induces Sleep, Behavioral, Neuroanatomical, and Neurophysiological Changes in Mice
Source: PLoS One. 2015 Dec 2;10(12):e0143909. doi: 10.1371/journal.pone.0143909 (PMC4668096; doi:10.1371/journal.pone.0143909)

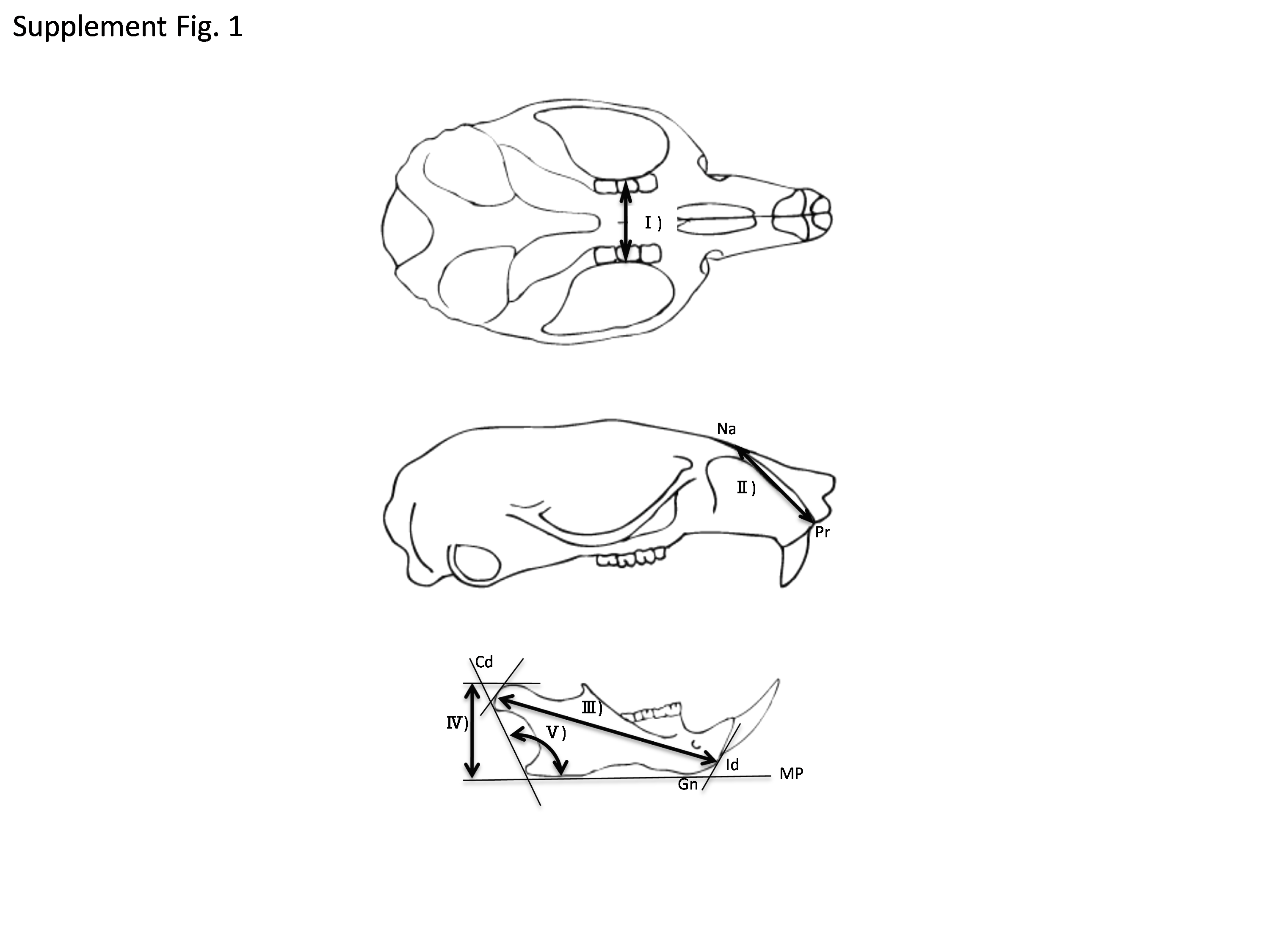

Supplement: S1 Fig — Nasion (Na): a point on the nasofrontal suture, Prosthion (Pr): the most inferior and anterior point on the alveolar process of the premaxilla, Condylion (Cd): most superodorsal point of the condylar head, Infradentable (Id): highest and most prominent point on the lower alveolar arch. Gnathion (Gn): lowest point on the mandiblar arch, Mandibular Plane (MP): Line tangent to the lower border of mandible through the Gn. Measurement items: I) length of buccal alveolar (between the maxillary second molar), II) length of maxilla (Na-Pr), III) length of mandible (Id-Cd), IV) ramus height (length of the vertical line to the MP from the highest point of condyle), V) gonial angle (the angle between MP and the line tangent to the most posterior border of mandibule through the Cd). (TIF) [file pone.0143909.s001.TIF]

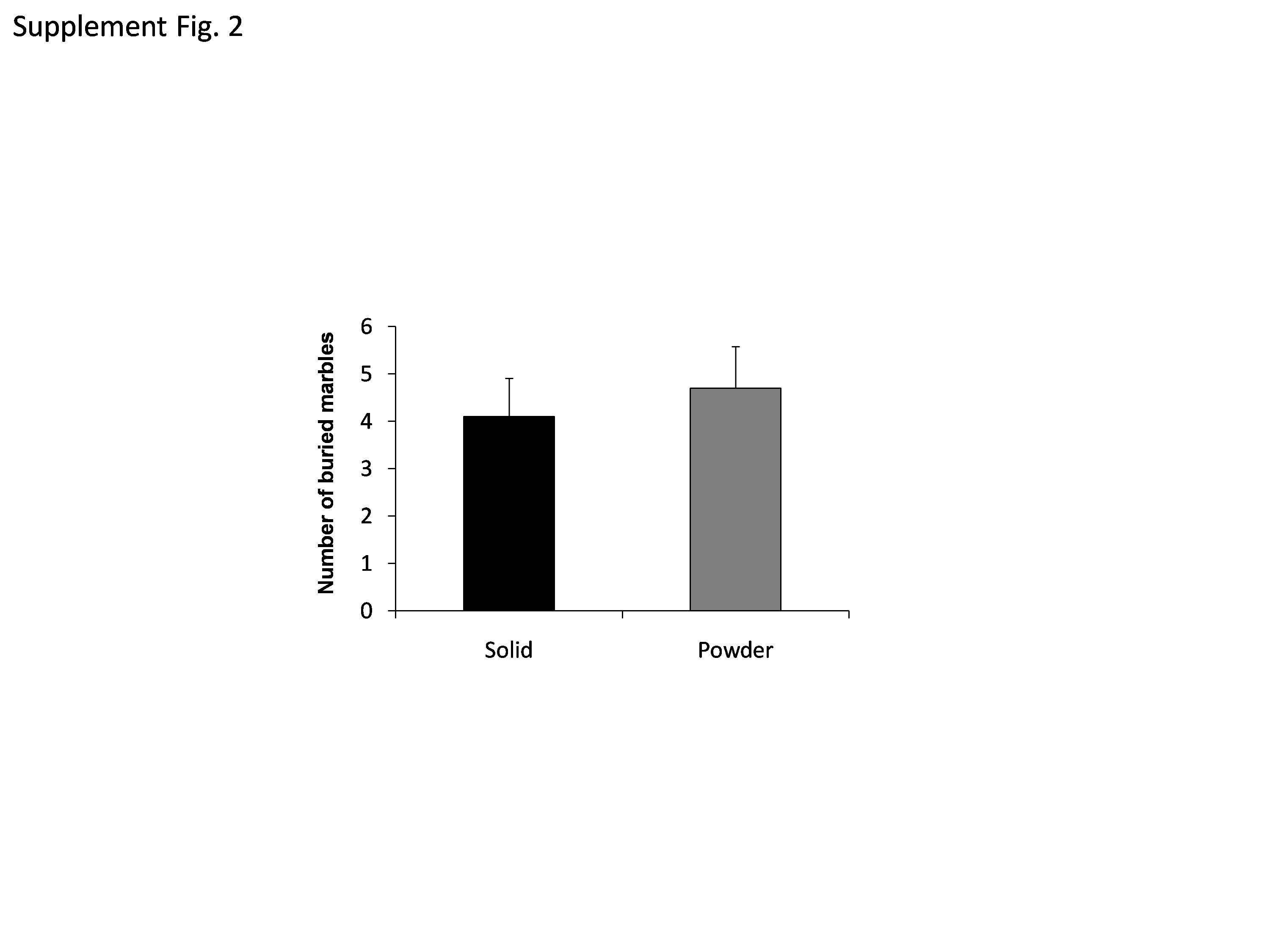

Supplement: S2 Fig — The marble burying test was performed before the EEG/EMG surgery at 18 weeks-old (N = 10 for each group). Data are presented as mean ± SEM. (TIF) [file pone.0143909.s002.TIF]
